# PET Imaging Radiotracers of Chemokine Receptors

**DOI:** 10.3390/molecules26175174

**Published:** 2021-08-26

**Authors:** Santosh R. Alluri, Yusuke Higashi, Kun-Eek Kil

**Affiliations:** 1 University of Missouri Research Reactor, University of Missouri, Columbia, MO 65211, USA; santoshreddy.alluri@yale.edu; 2 Department of Medicine, Tulane University, New Orleans, LA 70112, USA; yhigashi@tulane.edu; 3 Department of Veterinary Medicine and Surgery, University of Missouri, Columbia, MO 65211, USA

**Keywords:** chemokine receptor, chemokine, positron emission tomography (PET), in vivo imaging

## Abstract

Chemokines and chemokine receptors have been recognized as critical signal components that maintain the physiological functions of various cells, particularly the immune cells. The signals of chemokines/chemokine receptors guide various leukocytes to respond to inflammatory reactions and infectious agents. Many chemokine receptors play supportive roles in the differentiation, proliferation, angiogenesis, and metastasis of diverse tumor cells. In addition, the signaling functions of a few chemokine receptors are associated with cardiac, pulmonary, and brain disorders. Over the years, numerous promising molecules ranging from small molecules to short peptides and antibodies have been developed to study the role of chemokine receptors in healthy states and diseased states. These drug-like candidates are in turn exploited as radiolabeled probes for the imaging of chemokine receptors using noninvasive in vivo imaging, such as positron emission tomography (PET). Recent advances in the development of radiotracers for various chemokine receptors, particularly of CXCR4, CCR2, and CCR5, shed new light on chemokine-related cancer and cardiovascular research and the subsequent drug development. Here, we present the recent progress in PET radiotracer development for imaging of various chemokine receptors.

## 1. Introduction

Chemokines are biologically active peptides whose primary function is to induce cell migration. Chemokines share structural characteristics as they are all 8–10 kDa in mass and have two or four cysteine residues in the conserved domain. Based on the spacing of the first two cysteine residues, four major families of chemokines have been identified, which are CC, CXC, CX_3_C, and XC chemokines. The CC chemokines have two adjacent cysteines at the *N-*terminus, whereas the two *N-*terminal cysteine residues are separated by one and three amino acids in CXC and CX_3_C chemokines, respectively. Unlike these three, XC chemokines have only one *N-*terminal cysteine and another cysteine along with the downstream [1,2]. About 50 chemokine family members have been discovered, and the majority of them are represented by CC and CXC. Chemokines are involved in a number of biological processes and signal through binding to their specific cell surface receptors called chemokine receptors. Four subfamilies of chemokine receptors (CCR, CXCR, CX_3_CR, and XCR) are known based on their binding of specific chemokines. So far, 20 distinct chemokine receptors, 11 receptors for the CC chemokines (CCR 1–11), seven receptors for the CXC chemokines (CXCR 1–7), and one each for CX_3_C (CX_3_CR1) and XC (XCR1) chemokines, are characterized [2,3]. Table 1 briefly illustrates the significant chemokine receptors, their known specific chemokines, and their key immunoregulatory functions and associated diseases. All these receptors are G-protein coupled receptors (GPCRs) and mediate chemokine functions on their target cells. Protein kinase activation and intracellular Ca^2+^ mobilization are the major outcomes of chemokine receptor activation that exerts cellular responses, such as chemotaxis. Altogether, chemokines and their receptors are indispensable for homeostasis and functions of the immune system. Recent publications prompt that they also play a critical role in the progression of various cancers, cardiovascular diseases, autoimmune diseases, and neuroinflammation [3].

Functional imaging techniques using chemokine receptors have demonstrated their versatility in understanding the corresponding molecular pathways involved in disease, for disease monitoring, and identifying the underlying mechanisms of the diseases. Positron emission tomography (PET) is a valuable technique that provides essential insights into biological processes in living subjects. To date, several PET radiotracers have been developed to image different chemokine receptors in animal and human subjects. This review is intended to provide an overview of the development of various PET radiotracers for imaging of chemokine receptors involved in cancer and cardiovascular and pulmonary disorders.

### An Overview of Radiotracer Design

Of the radiotracers developed for chemokine receptors thus far, the majority of them are based on peptides and cyclam chelator moieties, and only a few of them are based on small molecules. Positron-emitting radioisotopes such as copper-64 (Cu-64, ^64^Cu, *t_1/2_* 12.7 h), gallium-68 (Ga-68, ^68^Ga, *t_1/2_* 67.7 min), fluorine-18 (F-18, ^18^F, *t_1/2_* 109.8 min), and carbon-11 (C-11, ^11^C, *t_1/2_* 20.4 min), have been employed to make these radiotracers. Irrespective of the radiotracer type, an ideal radiotracer should possess high affinity and specificity to its receptor and should have relatively fast clearance to allow low uptake in background tissues. In addition, an optimal radiotracer needs to present various radiochemical, biological, and clinical traits [64,65,66,67]. The radiochemical traits, such as radiotracer synthesis time (should be as short as possible), radiochemical yield (should be as high as possible), radiochemical purity (should be > 95%), and high molar activity play a crucial role prior to conducting biological experiments. Then, the in vitro and animal studies provide an initial validation of radiotracer’s stability or metabolism, cell uptake, receptor specificity and selectivity, binding activity, biodistribution, toxicity, receptor engagement in diseases, and its quantification. Finally, the first in human studies determine the radiotracer’s dosimetry, safety, reliability, and suitability as a clinical tracer to be used on a regular basis for suitable patients [64,68].

So far, small-molecule radiotracers have gathered the least consideration compared to peptidic or chelator-functionalized radiotracers for the imaging of chemokine receptors. One major disadvantage of peptidic radiotracers is their susceptibility to undergo degradation in the presence of endogenous enzymes, which results in lower metabolic stability. The large structures usually need to be chelated with radiometals, such as Cu-64 and Ga-68, towards the final radiotracer [69]. A good aspect of the chelation step is that it is generally faster compared to radiolabeling of a small molecule by, for example, F-18, but might require harsh radiolabeling conditions at the same time. The long half-life of Cu-64 allows the scanning of subjects for several hours after radiotracer injection, during which the nonspecifically bound radiotracer is cleared from the body, and high contrast images can be obtained. However, transchelation in tissues may be observed after tracer administration [70], and Cu-64 decays by emission of only 18% positrons (β^+^) and 43% by ionizing β-radiation, which may reduce the imaging quality. An alternative to Cu-64 is Ga-68, which has 89% of positron decay that offers good imaging efficiency [67,71]. However, the image resolution is relatively low because of the higher positron energy compared to that of F-18 [72].

Extensive arrays of small-molecule antagonists for various chemokine receptors have been developed over time. Notably, more than a few of these were shown to bind to a single class of chemokine receptors and have displayed their antagonism (*IC_50_* or *K_i_*) on chemokine–receptor interactions in the subnanomolar range [27,57,73,74,75]. High receptor specificity and affinity are desirable for an ideal PET radiotracer as the PET technique uses a trace amount of radiotracers. In addition, many of the reported small molecules allow to radiolabel them with C-11 or F-18 without changing their native structures. Changing the native structure for radiolabeling purposes can greatly influence the radiotracer’s affinity for its receptor. Nonetheless, radiometal labeling of a peptide or small protein that structurally mimics a chemokine should also be considered where the small-molecule radiotracer fails to engage its receptor. In the following sections, the development of various PET radiotracers for and PET imaging of chemokine receptors at the preclinical and clinical levels are reviewed.

## 2. Chemokine Receptors and Their PET Radiotracers

Over the years, significant efforts have been made to identify the roles of chemokines and their receptors in cancer events, such as tumor angiogenesis, metastasis, and tumor cell survival. Some of these chemokine receptors have become potential therapeutic targets in cancer treatment [76,77,78,79]. The receptor-specific inhibitors were shown to be viable for cancer treatment in preclinical models, and a few of them have entered clinical trials. Furthermore, some of these receptors were shown to play an important role in the progression of cardiac and pulmonary disorders [80,81,82,83]. Chemokine receptor-specific PET radiotracers can evaluate the engagement of the chemokine receptors in disease, investigate drug–receptor interactions, and further aid treatment planning.

### 2.1. CCR2 PET Radiotracers 

The monocyte chemoattractant protein-1 or CCL2 is the main interacting chemokine of the CCR2 receptor. Their interaction mediates calcium mobilization and inhibition of adenyl cyclase and is shown to be central to pain development in osteoarthritis and rheumatoid arthritis [9,10]. In addition, studies using CCR2-deficient mice models indicated that the receptors were involved in the development of Alzheimer’s-like pathology and obesity [13]. Furthermore, changes in CCR2 expression were noted in cardiac and pulmonary disorder models and, to some extent, in cancer models such as adenocarcinoma [84,85]. Blockade of CCR2 was demonstrated to be viable in treating various inflammatory diseases, experimentally and clinically [86,87]. A CCR2-selective PET radiotracer would be an addition to CCR2-related research and give the possibility to investigate CCR2 engagement in certain diseases.

**Figure 1:**
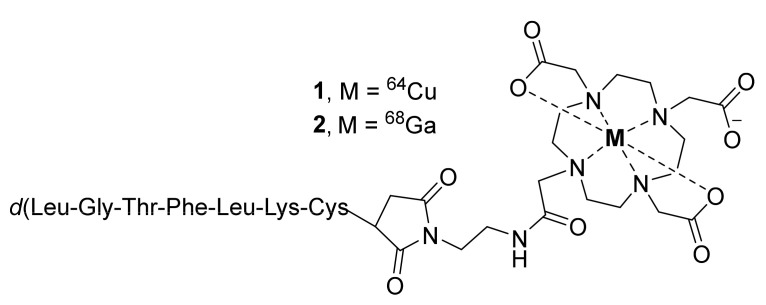
PET radiotracers of CCR2.

In search of a potent CCR2 antagonist, Auvynet et al., in 2016 have developed a short peptide consisting of seven d-amino acids (LGTFLKC) and named it extracellular loop 1 inverso (ECL1i). The in vitro and in vivo studies using ECL1i demonstrated its allosteric antagonism, selectivity, and potency against CCR2 [88]. Based on this peptide, Liu et al., in the same year reported ^64^Cu-radiolabeled ECL1i conjugated to DOTA (Figure 1, [^64^Cu]DOTA-ECL1i, **1**) to image CCR2-positive cells in ischemia-reperfusion injury after lung transplantation in wild-type and CCR2-deficient mice by PET [86]. The lungs were transplanted after 18 h of cold ischemia. The study also used a ^64^Cu-doped gold nanocluster conjugated to ECL1i ([^64^Cu]AuNCs-ECL1i) for improved detection of CCR2. Both tracers were shown to be specific to CCR2^+^ cells, wherein the PET blocking studies were performed with ECL1i. PET biodistribution studies of both tracers were assessed one hour after lung transplantation. Radiotracer **1** displayed high uptake in the kidneys and minor accumulation in the liver and spleen. Interestingly, its uptake was noted to be higher in the donor’s lungs than in the native, which suggested the accumulation of inflammatory cells into lung grafts. Likewise, after the transplantation, elevated CCR2 expression was noted throughout the recipient’s body, with the highest expression in lung grafts. The multivalent gold-conjugated tracer was shown to improve imaging efficiency through extended blood circulation at one hour and significant renal accumulation.

In the following year, the same group used **1** to characterize CCR2 in mouse models of lung injury (PET study) and in human tissues (autoradiography study) from subjects with severe chronic obstructive pulmonary disease (COPD) [87]. Lung inflammation in both wild-type and CCR2-deficient mice was induced by the administration of lipopolysaccharide (LPS), and the control mice were administered with saline. Overall, radiotracer **1** displayed CCR2-specific signals with rapid blood clearance (<1% ID/g at 1 h post-injection (p.i.)) in mice, indicating the requirement of chemical modifications to improve its pharmacokinetic properties. The authors also stated that clinical studies might be required to validate the increased binding signal from **1** in the lungs of COPD subjects. The same group again in 2019 described a Ga-68-labeled ECL1i conjugated to DOTA ([^68^Ga]DOTA-ECL1i, **2**) to visualize CCR2-positive monocytes and macrophages in mouse models of cardiac injury (diphtheria toxin-induced cardiomyocyte ablation and ischemia-reperfusion myocardial injury) [89]. PET biodistribution studies demonstrated the excellent myocardial uptake of **2** and its specificity to CCR2 within the heart regions. The authors pointed out that in addition to monocytes and macrophages, radiotracer **2** may also identify other immune cells such as dendritic cells and lymphocytes that express CCR2. Therefore, a detailed understanding of immune composition is needed to elucidate the cell specificity of **2**.

Recently, the same group came up with ^64^Cu-labeled copper nanoparticles ([^64^Cu]Cu@CuO_x_) conjugated to ECL1i or to a combination of ECL1i and gemcitabine (Gem) and applied them in mice models of pancreatic ductal adenocarcinoma (PDAC) [90]. The radiotracer [^64^Cu]Cu@CuO_x_-ECL1i (**3**) displayed high specific uptake in tumor and low nonspecific retention in comparison to those of 2-[^18^F]fluoro-2-deoxyglucose ([^18^F]FDG). This signifies the tracer’s potential in the early detection of PDAC malignancy. Additionally, treatment with Cu@CuO_x_-ECLi-Gem led to tumor necrosis and extended the survival of mice. The authors indicated that further studies were required to validate these nanoparticles as image-guided therapeutic agents.

### 2.2. CCR5 PET Radiotracers

As stated above, CCR5 receptor expression was found on various immune cells such as monocytes, macrophages, neutrophils, T lymphocytes, dendritic cells (DCs), and natural killer (NK) cells. It recruits these immune cells to the sites of infection and inflammation by interacting with various chemokines, such as CCL3, CCL4, CCL5, CCL8, and CCL13 [3]. Moreover, CCR5 serves as a critical coreceptor along with CXCR4 for human immunodeficiency virus (HIV) entry into CD4^+^ immune cells. The gp120 envelope protein of HIV binds to these chemokine receptors after anchoring the CD4^+^ cells [91,92]. CCR5 is also shown to regulate the chemotaxis of neutrophils and monocytes in the early progression and late stage of atherosclerosis [93,94]. Furthermore, CCR5 is shown to mobilize tumor-associated macrophages (TAM) [95], myeloid-derived suppressor cells (MDSC) [96], and regulatory T (T_reg_) lymphocytes [97]. In tumor models, these cells promote the growth of various tumors by suppressing immune systems or by exerting protumor activity through immuno-suppressive agents such as interleukin-10 (IL-10) and transforming growth factor-β (TGF-β). TAM and MDSC also produce various growth factors, such as vascular endothelial growth factor (VEGF) and fibroblast growth factors (FGF), that stimulate the proliferation, metastasis, and angiogenesis of tumor cells [98,99]. On the other hand, CCR5 is also one of the critical controllers of tumor-infiltrating lymphocytes (TILs), such as cytotoxic T lymphocytes (CTLs) that inhibit tumor growth [100]. Given its involvement in infectious diseases, cancer, and cardiac disorders, CCR5-PET radiotracers are important to fully understand the role of CCR5 in certain diseases and subsequent drug development.

**Figure 2:**
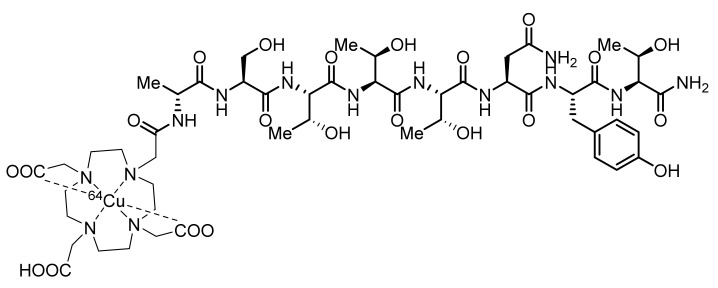
Chemical structure of [^64^Cu]DOTA-DAPTA (**4**) for CCR5 imaging.

The development of a CCR5 radiotracer has been limited, and tracers for this target have only been applied in mouse models of atherosclerosis. d-Ala1-peptide T-amide (DAPTA) is a synthetic CCR5-specific octapeptide (d-Ala-Ser-Thr-Thr-Thr-Asn-Tyr-Thr-NH_2_) that inhibits the entry of HIV with 0.1 nM antagonist activity [101]. In 2014, Luehmann et al., developed a Cu-64-labeled DOTA-DAPTA ([^64^Cu]DOTA-DAPTA, 4, Figure 2) and a Cu-64-labeled DOTA-DAPTA-comb nanoparticle (**5**) [102,103]. The DOTA-DAPTA-comb nanoparticle is a block copolymer of poly(methacrylate) whose ester groups were conjugated to PEG and PEG-DAPTA. PET studies were performed in the vascular injury model of apolipoprotein E (ApoE) knockout (KO) mice with a high-fat diet. Both **4** and **5** displayed specific uptake in injury lesions, but **5** showed better signals in the injured artery and overall better pharmacokinetics than **4**, which displayed fast clearance from the blood. The high retention of **5** in blood was optimized by changing the conjugating composition of DAPTA (10%, 25%, and 40%) in the comb nanoparticle. The authors demonstrated that the 40% [^64^Cu]DAPTA-Comb was shown to offer good sensitivity and selectivity for the imaging of CCR5 upregulation in atherosclerotic plaque. Some more studies are to be expected in the near future for further validation and translation of **5**. On the other hand, given the availability of a plethora of CCR5 antagonists, small-molecule radiotracer development that could contribute to the above findings still needs to be pursued.

### 2.3. CCR8 PET Radiotracers

**Figure 3:**
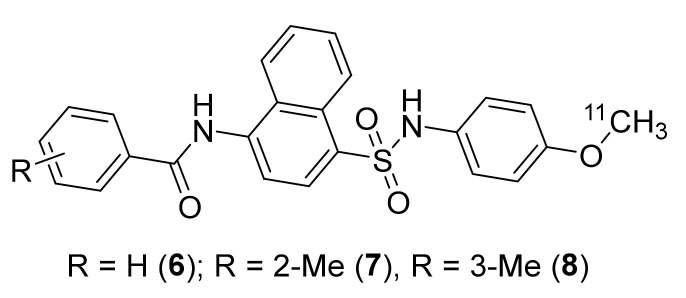
PET radiotracers of CCR8.

The expression of CCR8 has been found on various cell types, including T-helper-2 (Th2) cells, microglial cells, and monocytes. The CCR8–CCL1 interaction is suspected of playing a role in CCR8 expression changes in diseases such as multiple sclerosis [104]. However, more functional studies are required to validate the role of CCR8 in certain diseases. In 2007, a few CCR8-selective and potent antagonists based on a naphthalene-sulfonamide core were developed using human CCR8 in vitro assays [74,104]. Based on this, Wang et al., in 2008 developed [^11^C]**6** (CCR8 *Ki* 0.17 ± 0.05 nM), [^11^C]**7** (CCR8 *Ki* 1.6 ± 0.07 nM), and [^11^C]**8** (CCR8 *Ki* 42.6 ± 2.8 nM) with molar activity (MA) ranging 74–111 GBq/μmol at the end of bombardment (EOB) (Figure 3) [105]. Radiotracers **6**, **7,** and **8** were prepared using C-11 methylation from the corresponding *N*-desmethyl and *O-*desmethyl precursors, respectively. However, no PET imaging studies using either of these tracers were reported.

### 2.4. CXCR4 Receptor and PET Tracer

Upregulation of CXCR4 has been noted in various cancers, including breast cancer, gastric cancer, non-small cell lung cancer (NSCLC), and renal cell carcinoma. Furthermore, overexpression of CXCR4 in tumor tissues was shown to correlate with tumor aggressiveness and elevated risks of metastasis and recurrence [75,106]. Much of the PET imaging of CXCR4 has already been reviewed [64,68,107]. A brief emphasis on the previous works and recent developments in CXCR4-PET are presented in this review. CXCR4 is undoubtedly a widely explored chemokine receptor with respect to cancer imaging. It plays a key role in tumor growth and metastasis in various types of human cancers and has become a subject of intensive research. As cited above, CXCR4 along with CCR5 works as a coreceptor for HIV transmission and disease. The early antagonists for the CXCR4 receptor were mainly based on peptides, which were indeed referred to as HIV-1 entry inhibitors [108,109]. Furthermore, crystal structure-based structure–activity relationship (SAR) studies led to the discovery of several promising small-to-large peptides and also small-molecule antagonists for CXCR4.

**Figure 4:**
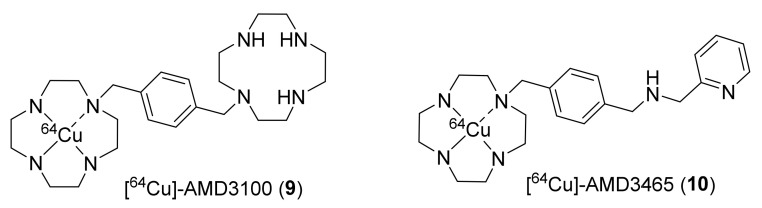
Early PET radiotracers of CXCR4.

During the late 1990s, an anti-HIV research program facilitated the discovery of bicyclam AMD-3100, which was shown to have anti-HIV activity through partial inhibition of CXCR4 activity [108]. Based on this, Nimmagadda et al., in 2009 have developed [^64^Cu]AMD3100 (**9**) for the imaging of CXCR4 in mice (Figure 4) [110]. Radiotracer **9** was prepared from [^64^Cu]acetate and AMD-3100 with a molar activity (MA) of 418 GBq/μmol at the end of synthesis (EOS). Biodistribution studies using **9** showed rapid clearance from blood and indicated its specific accumulation in CXCR4-expressing organs, such as bone marrow and spleen. The specific binding of **9** was further confirmed via blocking studies with CXCR4 ligand Stomal-cell Derived Factor-1 (SDF-1 or CXCL12). The major limitation of **9** was its high accumulation in the liver (≥40% ID/g), which masked some adjacent organs. Radiotracer **9** was then utilized in a couple of other studies to image CXCR4 in tumor models, such as Waldenström Macroglobulinemia, brain tumor xenografts, and lung metastases derived from MDA-MB-231 breast cancer cells [111]. The authors revealed that **9** presented good properties as a PET radiotracer to image CXCR4. However, it had relatively low affinity (~0.651 ± 0.037 μM), large size, high overall positive charge, and restricted structure scaffold.

To overcome this, the same group in 2011 employed monocyclam-based AMD3465, which was shown to have stronger and specific affinity for CXCR4 than AMD3100 and reduced charge and size [112,113,114]. The group successfully synthesized [^64^Cu]AMD3465 (**10**) using [^64^Cu]copper(II) chloride and AMD3465·6HCl with a MA of 6.0 ± 3.1 GBq/μmol at the EOS (Figure 4). PET studies were performed in mice bearing CXCR4-expressing U87 brain tumors and HT-29 colon tumors. Biodistribution studies indicated high CXCR4-specific uptake in tumors in both models. It demonstrated higher tumor-to-muscle and tumor-to-blood ratios (from seven- to eight-fold higher) at 90 min p.i. in comparison to those of radiotracer **9**. Significant uptake of **10** in the liver (≥32% ID/g) and kidney was also observed, similar to that of **9**. The tracer was shown to have overall good kinetics and the potential to be exploited in clinical studies for the detection of CXCR4 expression in human cancers [115,116].

**Figure 5:**
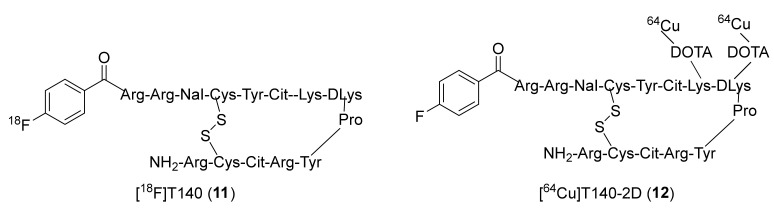
TN-14003 based PET radiotracers of CXCR4.

The next class of CXCR4-PET radiotracers was based on cyclic peptide CXCR4 antagonists. Many efforts have been put towards this class of PET radiotracers and generated interest from the radiochemistry community. In 2010, Chen’s group utilized 4-fluorobenzoyl-TN14003 (4-F-T140), a tetradecapeptide CXCR4-selective antagonist, to prepare [^18^F]4-F-T140 (**11**, Figure 5) [117]. This antagonist has an IC_50_ value of 0.8 nM against CXCR4 and is currently under clinical investigation for the treatment of various malignancies and solid tumors. The radiotracer **11** was synthesized in three steps over 90 min, starting from ^18^F-fluoride to generate *N*-succinimidyl-4-[^18^F]fluorobenzoate intermediate, which was subsequently conjugated to T140 peptide. The reported MA of **11** was 7 ± 2 GBq/μmol at the EOS. PET studies using **11** were performed in mice bearing Chinese hamster ovarian (CHO)-CXCR4 and CHO tumors. It presented good specific uptake in CXCR4-expressing organs, such as the spleen (12 ± 2% ID/g at 3 h p.i.) and bone marrow (2.6 ± 0.65% ID/g at 3 h p.i.). It was also shown to have CXCR4-independent and undesired binding to red blood cells. In addition, long radiosynthesis times and low radiochemical yields limited its use for CXCR4 imaging in tumors.

To overcome these issues, the same group in 2011 developed ^64^Cu-4-F-Benzoyl-T140 conjugated to two DOTA moieties and named [^64^Cu]T410-2D (**12**, Figure 5) [118]. PET studies were performed using **12** in mice similar to that of **11.** Though the tracer was shown to image CXCR4-positive tumors and displayed CXCR4-specific uptake in the spleen (16.15 ± 1.69% ID/g) and bone marrow (9.45 ± 1.53% ID/g), it had high undesired accumulation in the liver (16.43 ± 1.40% ID/g), kidneys (40.40 ± 7.25% ID/g), and red blood cells (RBCs, 12.73 ± 0.23% ID/g) at 4 h p.i.

**Figure 6:**
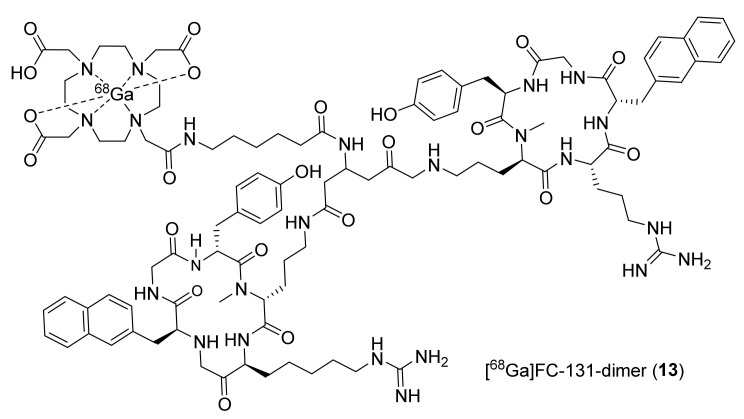
Dimerized FC-131 PET radiotracer of CXCR4.

During the same timeline, Demmer et al., utilized a dimeric peptide (FC131) conjugated to DOTA and labeled it with Ga-68 to synthesize **13** (Figure 6). This dimer was shown to have high affinity for CXCR4 (IC_50_ 39 ± 2 nM), low toxicity, and high stability toward enzymatic degradation, which is typically associated with cyclic pentapeptides [119,120,121]. Biodistribution studies were performed using **13** in mice bearing human OH-1 small cell lung cancer (OH1 h-SCLC) tumors. It displayed the highest accumulation in the liver (44.3 ± 5.5% ID/g), lungs (2.1 ± 0.3% ID/g), and spleen (4.0 ± 0.6% ID/g) at 1 h p.i. Though its tumor accumulation (2.1 ± 0.5% ID/g) is limited, it was notably higher compared to those of blood (1.9 ± 0.3% ID/g) and muscle (0.4 ± 0.1% ID/g) at 1 h p.i. The authors indicated that the dimer had high lipophilicity, due to which **13** displayed suboptimal biodistribution.

**Figure 7:**
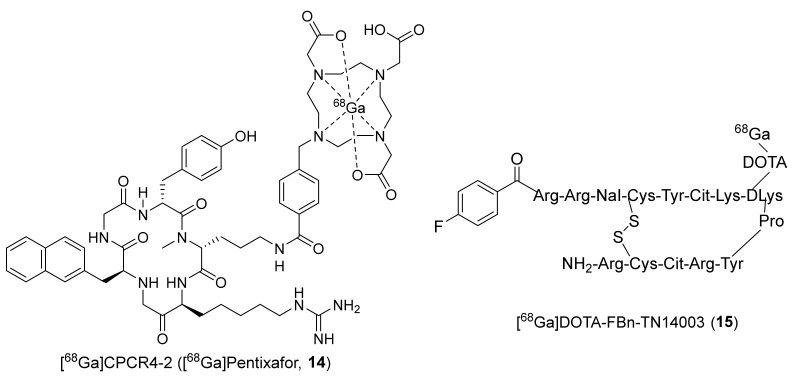
Ga-68-labeled pentixafor (CPCR4-2, **14**) and TN14003 (**15**).

The same research group evaluated the monomeric form of FC131 (CPCR4.2 or Pentixafor) conjugated to DOTA and labeled it again with Ga-68 to synthesize [^68^Ga]pentixafor (**14**, Figure 7) [122,123]. The reported IC_50_ value of this ligand was 5 ± 1 nM against CXCR4 expressing Jurkat cells. The radiotracer **14** displayed excellent biodistribution in mice bearing OH-1 h-SCLC tumors with higher uptake in tumors (6.16 ± 1.16% ID/g) and significantly lower uptake in the liver (1.85 ± 0.24% ID/g) at 1 h p.i. The tumor-to-blood (5.8 ± 0.9) and tumor-to-muscle (16.6 ± 3.8) ratios for **14** were also considerably higher than those found for other peptidic CXCR4 radiotracers. This radiotracer was translated further to several clinical studies (vide infra) for the imaging of CXCR4 in various tumors.

Likewise, Hennrich et al., in 2012 developed ^68^Ga-4-F-Benzoyl-T140 conjugated to DOTA ([^68^Ga]DOTA-FBn-TN14003, **15**¸ Figure 7) [124]. The IC_50_ value of nonradioactive standard **15** was determined against CXCR4, and the value was 1.99 ± 0.33 nM. The radiotracer was prepared with a good MA of 29.8 ± 3.1 GBq/μmol. The uptake studies of **15** were performed in vitro in Jurkat cells, and MDA-MB-231 cells, wherein stable uptake was observed in MDA-MB-231 cells, and a decreased uptake was observed in Jurkat cells at 1.5 h, suggesting fast kinetics to be expected for a short peptide. However, no in vivo studies using **15** have been reported so far.

**Figure 8:**
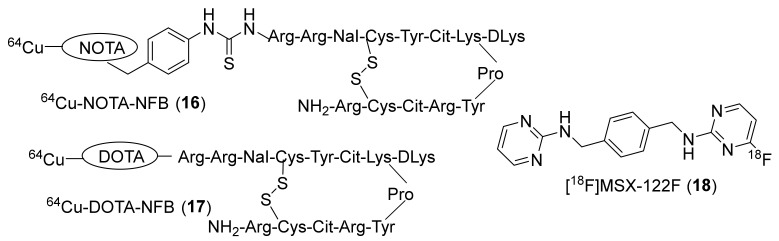
Chemical structures of [^64^Cu]NOTA-NFB (**16**), [^64^Cu]DOTA-NFB (**17**), and [^18^F]MSX-122F (**18**).

To limit the undesired accumulation of radiotracers **11** and **12** in RBCs, Chen’s group again in 2012 came up with two different peptides, wherein they replaced the fluorobenzoyl group of FBn-TN14003 with either NOTA or DOTA and labeled them with Cu-64 to prepare [^64^Cu]NOTA-NFB (**16**) and [^64^Cu]DOTA-NFB (**17**) (Figure 8) [125]. The syntheses of both of these radiotracers were achieved using [^64^Cu]acetate and the respective peptide precursors over 50–55 min with MA of 12.21–14.8 MBq/μg. PET studies were performed in mice bearing CXCR4-positive and CXCR4-negative CHO tumors. They observed specific uptake in CXCR4-positive tumors with 4.38 ± 0.68% ID/g and 3.58 ± 0.67% ID/g at 4 h p.i. for **16** and **17**, respectively. In addition, unlike **11** and **12**, both of these derivatized radiotracers showed no accumulation in blood and displayed high tumor-to-blood ratios at 4 h p.i. (38.88 ± 3.91 for **16**, 14.50 ± 0.82 for **17**).

In the same year, Liang et al., developed a unique small molecule (MSX-122) which was shown to have partial antagonist properties against CXCR4 (IC_50_ ~10 nM) [126]. The radiotracer [^18^F]MSX-122F (**18**, Figure 8) was prepared in one step through an aromatic nucleophilic (SnAr) substitution reaction using ^18^F-fluoride and the corresponding aryl-chloride precursor. The in vivo studies using MSX-122 specified its potency and its usefulness as an anti-inflammatory and antimetastatic agent in animal models. The in vitro functional assays using **18** indicated CXCR4 specific binding. However, no in vivo studies using **18** have been reported.

**Figure 9:**
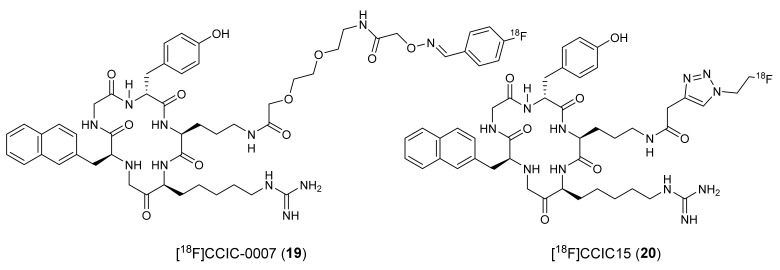
Chemical structures [^18^F]CCIC-0007 (**19**) and [^18^F]CCIC15 (**20**).

To develop ^18^F-fluoride-labeled peptidic CXCR4 radiotracers, Aberg et al., derivatized FC131 into two different analogs and synthesized [^18^F]CCIC-0007 (**19,** IC_50_ 0.80 μM) and [^18^F]CCIC15 (**20,** Figure 9) [127,128]. Radiotracer **19** was synthesized via bioconjugation of 4-[^18^F]fluoro-benzaldehyde and aminooxy-PEG-functionalized FC131, and **20** was synthesized through the click chemistry approach using 2-[^18^F]fluoroethylazide and a terminal alkyne functionalized FC131. The in vivo studies using **19** indicated low specific binding with high undesired accumulation in the elimination tissues. The in vitro studies using **20** showed high uptake in CXCR4 overexpressing U87·CD4 cells and low uptake in CXCR4-negative cells. However, no further studies using either of these two radiotracers were reported.

**Figure 10:**
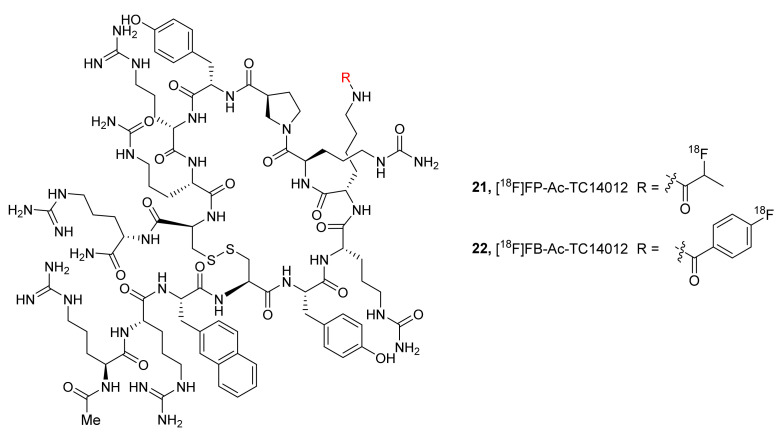
F-18-labeled radiotracers based on CXCR4 inverse agonists, **21** (R = 2-[^18^F]fluoropropionyl) and **22** (R = 4-[^18^F]fluorobenzoyl).

In 2013, Zhang et al., have described two ^18^F-labeled radiotracers based on an Ac-TC14012 peptide, selective CXCR4 inverse agonist (IC_50_ 2.47 ± 0.53 nM). They synthesized 4-nitrophenyl-2-[^18^F]fluoropropionate ([^18^F]FP) and *N*-succinimidyl-4-[^18^F]fluorobenzoate ([^18^F]FB) and coupled them to Ac-TC14012 to prepare [^18^F]FP-Ac-TC14012 (**21**) and [^18^F]FB-Ac-TC14012 (**22**), respectively, with MA ranging from 18.7 to 31.6 GBq/μmol at the EOS (Figure 10) [129]. Cell uptake studies using both of the radiotracers were performed with CXCR4-transfected CHO cells, and PET studies were conducted in mice bearing subcutaneous CHO-CXCR4 and CHO cells. At 2 h p.i., radiotracer **21** was shown to display higher tumor uptake (4.30 ± 0.86% ID/g) than **22** (1.86 ± 0.19% ID/g), low nonspecific binding in tissues, and good tumor-to-background contrast. Though the CXCR4-expressing tumors could be visualized, both radiotracers also showed high uptakes in metabolic organs, similar to the previous peptidic CXCR4 radiotracers.

**Figure 11:**
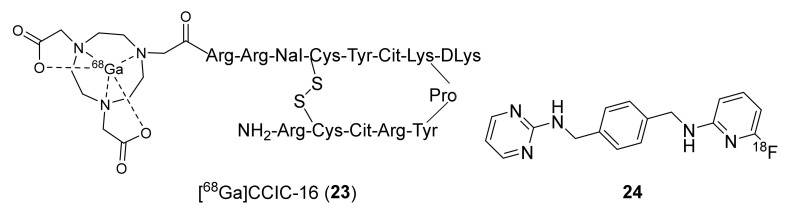
Structures of [^68^Ga]CCIC-16 (**23**) and small-molecule F-18 radiotracer (**24**).

Using the derivatization strategy at the *N-*terminus of TN14003 peptide, as in **16** and **17**, George et al., in 2014 designed and synthesized [^68^Ga]CCIC-16 (**23**, MA 2.76 ± 0.61 GBq/μmol) to selectively image CXCR4 in animal models using PET (Figure 11) [130]. Dynamic PET imaging studies using **23** were performed in mice bearing U87·CD4 or U87·CD4·CXCR4 tumor cells. The radiotracer displayed tumor uptake specific to CXCR4 expression with good tumor-to-muscle (9.5 ± 1.7) and tumor-to-blood (6.3 ± 1.8) ratios. Overall, it showed promising results with respect to CXCR4 imaging in tumor models, and further studies are needed to ascertain its reliability.

To develop a brain-penetrating CXCR4 PET radiotracer, Demoin et al., in 2016 described an ^18^F-labeled pyrimidine-pyridine agent (**24,** CXCR4 EC_50_ 1 nM, Figure 11) [131]. It was prepared through a conventional SnAr substitution reaction using [^18^F]fluoride and the corresponding aryl-nitro precursor. The authors concluded that radiotracer **24** displayed low cellular uptake in in vitro studies and rapid metabolism in in vivo studies, and further structural modifications are required to develop a suitable blood–brain barrier (BBB)-penetrating CXCR4 imaging probe. 

**Figure 12:**
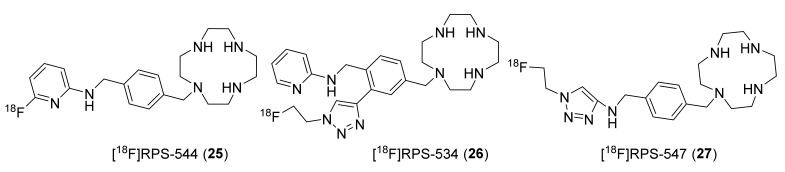
F-18-labeled CXCR4 radiotracers based on AMD-3465.

To improve CXCR4 specific detection in tumors, Coarssa et al., in 2018 reported AMD-3465 analogous [^18^F]RPS-544 (**25**, Figure 12) [132]. It was synthesized via a SnAr reaction involving [^18^F]fluoride and *-Boc*-protected NO_2_-AMD-3465 precursor with MA of >185 GBq/μmol. PET biodistribution studies in mice bearing PC3-CXCR4 tumors indicated good specific uptake of **25** in tumors (3.4 ± 1.2% ID/g) and significant uptake in the liver (15–25% ID/g) and kidneys (25–35% ID/g) at 1 h p.i. Further in silico screening by the same group led to fluoroethyltriazolyl monocyclam derivatives as CXCR4 antagonists. They chose six different compounds based on a high docking score and radiolabeled them via the click chemistry approach using [^18^F]fluoroethyl azide and corresponding alkyne precursors, with MA of >185 GBq/μmol at the EOS. Though CXCR4 affinity was low for these compounds, the radiotracers [^18^F]RPS-534 (**26**, RPS-534, IC_50_ 218 ± 38 nM) and [^18^F]RPS-547 (**27**¸ RPS-547, IC_50_ 601 ±118 nM) displayed good tumor-to-blood, tumor-to-muscle, and tumor-to-lung uptake ratios in PC3-CXCR4 xenograft tumors (Figure 12). More studies might be required to improve the utility of these tracers as CXCR4 imaging agents in tumors [133].

**Figure 13:**
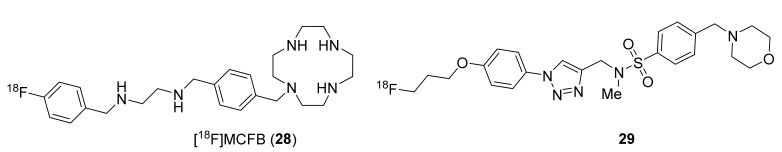
Recent PET radiotracers of CXCR4.

Considering the limitations of previous ^18^F-labeled cyclam derivatives, Brickute et al., in 2019 described a metabolically stable ^18^F-labeled radiotracer ([^18^F]MCFB, **28**, Figure 13) based on AMD-3465, wherein they replaced 2-pyridylmethylamine of AMD-3465 with 1-aminomethyl-4-fluorobenzene [134]. The radiosynthesis of **28** was accomplished via reductive amination using 4-[^18^F]fluorobenzaldehyde and the corresponding primary amine precursor with a modest MA of 5.7 GBq/μmol. The authors concluded that **28** is not a suitable CXCR4 probe in tumors owing to its high accumulation in the liver. 

A very recent effort by Oum et al., resulted in a small molecular CXCR4 targeted ^18^F-labeled benzenesulfonamide derivative (**29**, Figure 13). It was synthesized through a nucleophilic substitution reaction using ^18^F-fluoride and the corresponding mesylate precursor [135]. Though multiple studies are required, this radiotracer was shown to have potential in tumor imaging and displayed good uptake in human tumor xenografts, metastatic lung tumor tissue of mice, and inflammatory lesions.

**Figure 14:**
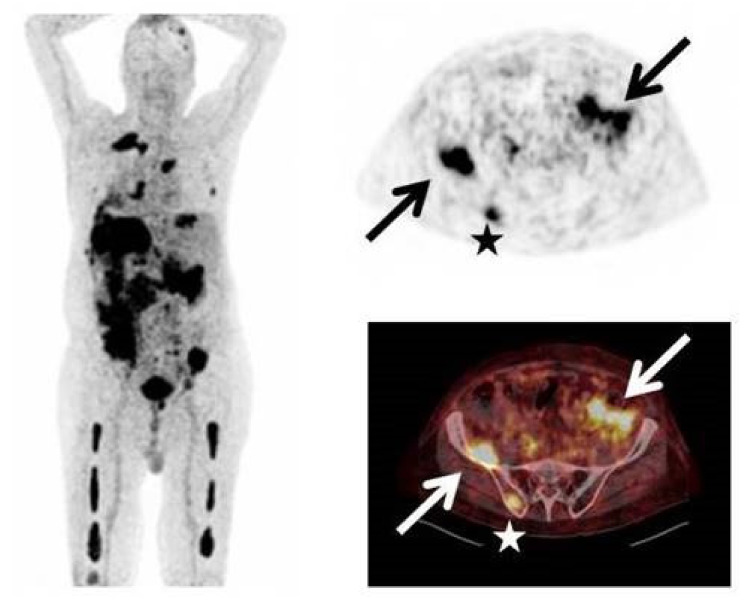
Intense uptake of **14** in a patient with multiple myeloma (type Ig A λ) and rising free serum light chains (*Left*). Stars represent uptake of **14** in multiple intramedullary lesions, arrows represent uptake in extramedullary lesions (*Right*). Adapted from an open access publication by Lapa et al. [137].

Currently, [^68^Ga]pentixafor (**14**) serves as an important PET radiotracer for CXCR4-specific detection at the clinical level to diagnose certain cancers such as multiple myeloma and glioma. For instance, in 2016, a clinical study was conducted using **14** in 15 patients with glioma to enable the detection of CXCR4, and the imaging results were compared with that of *O*-(2-^18^F-fluoroethyl)-L-tyrosine ([^18^F]FET)-PET [136]. The study reported CXCR4-positive disease in 13 subjects with standard uptake value (SUV)_mean_ and SUV_max_ of 3.0 ± 1.5 and 3.9 ± 2.0, respectively, for **14** and 4.4 ± 2.0 and 5.3 ± 2.3 for [^18^F]FET. The tumor-to-background ratios for **14** (70.3 ± 44.0 at SUV_max_) were observed to be higher than those for ^18^F-FET (3.8 ± 1.2). Likewise, in 2017, Lapa et al., conducted a clinical study in 35 patients with multiple myeloma (MM) for PET imaging of CXCR4 (Figure 14), and the results were compared with those of [^18^F]FDG [137]. 

The study showed that **14** enabled CXCR4-positive detection in 23 subjects and also detected previously unknown myeloma lesions in 21% of subjects. Recently, Li et al., conducted a similar study in 30 patients using **14** for assessment of newly diagnosed MM and indicated that **14** showed a higher positive rate than [^18^F]FDG in the chosen patient population [138]. Amongst the chemokine receptors, the CXCR4 receptor is undoubtedly a potential therapeutic target in the treatment of certain cancers [139,140]. In addition, the receptor was also shown to play a significant role in the progression of atherosclerosis plaques [141,142]. As indicated above, several efforts have been made to develop various PET radiotracers (peptidic, cyclam) for CXCR4 imaging. The PET radiotracer development with small-molecule CXCR4 inhibitors has so far garnered less attention. Several small molecules have been and are being reported as CXCR4 antagonists with inhibitory concentrations in the subnanomolar range. With the advantage of radiolabeling of small-molecule CXCR4 antagonists with either F-18 or C-11 radioisotopes, potential PET radiotracers specific to CXCR4 can be expected in the near future.

### 2.5. CXCR7 Receptor and PET Radiotracer

Studies suggest that the overexpression of the CXCR7 (or ACKR3) receptor has been found in various cancers and has been shown to modulate the tumor microenvironment and tumor cell survival. Notably, small-molecule CXCR7 inhibitors were shown to suppress tumor growth in mouse models of breast and lung cancer [143]. PET radiotracer development for this receptor is still in the early stages. To provide direct visualization of ACKR3 expression in vivo, Nimmagadda’s group reported an ACKR3-targeted monoclonal antibody radiolabeled with ^89^Zr ([^89^Zr]ACKR3-mAb, **30**) [144]. PET studies using **30** were performed in mice bearing 231-ACKR3, MCF-7, KYSE520 (esophageal), and HCC95 (lung) tumor cells. They also performed in vitro assays to correlate the cell surface CXCR7 expression in tumor cells with the data gathered from Cancer Cell Line Encyclopedia, The Cancer Genome Atlas. PET studies indicated CXCR7-specific uptake of **30** in all the tumor xenografts, and in vivo blocking study further confirmed the specific uptake. Significant uptake of **30** was also observed in the spleen and was noted to be partially due to the CXCR7 expression in splenic marginal B-cells and splenic venous endothelial cells. The clearance of nonspecific uptake in the liver and spleen was observed after 120 h p.i. of **30**. Overall, the radiotracer has shown a promise in selective imaging of CXCR7 expression in different tumor models, and that CXCR7 is an important biomarker for noninvasive imaging of CXCR7-overexpressing malignancies. However, no further studies using **30** have been reported.

### 2.6. CX_3_CR1 PET Radiotracers

CX_3_CL1 is a sole member of the CX_3_C family and is also called Fractalkine (FKN). CX3CR1 is a cognate receptor of CX3CL1. The expression of CX_3_CR1 has been found in the brain, spleen, and on various cell types such as monocytes, microglia, macrophages, and neutrophils. Cell studies indicate that the CX_3_CR1 receptor plays an important role in the migration and adhesion of lymphocytes and leukocytes during an inflammatory response [60,61,62,63]. Furthermore, its expression in microglia was shown to be upregulated in murine models of experimental autoimmune encephalomyelitis (EAE), which suggests it might be important during brain inflammatory responses. Although additional studies are required to comprehend its role in peripheral regions, CX_3_CR1 became one of the neuroprotective targets. CX_3_CR1 specific antagonists are important to study the changes of CX_3_CR1 expression upon binding of CX_3_CL1 during an inflammatory response in the brain and to understand the roles of downstream signaling pathways.

**Figure 15:**
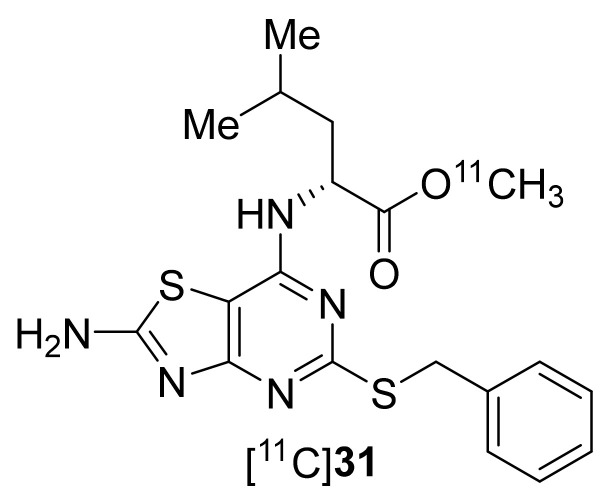
C-11-labeled radiotracer of CX3CR1.

Around the year 2013, AstraZeneca had developed a series of CX_3_CR1-selective antagonists based on a 7-Amino-5-thio-thiazolo[4,5-*d*]pyrimidine core, using SAR studies and *h*CX_3_CR1 cell assays [63]. Based on this, Gao et al., in 2017 prepared **31** with MA 370–1110 GBq/μmol at the EOB (Figure 15) [145]. Radiotracer **31** was prepared using C-11 methylation from the corresponding carboxylic acid precursor. However, the tracer displayed low CX_3_CR1 binding affinity and high nonspecific binding in saturation and competitive binding assays and was concluded as not suitable for PET imaging of CX_3_CR1 receptors.

## 3. Conclusions

There has been great progress towards the development of PET radiotracers for various chemokine receptors. Promising results from PET studies using [^68^Ga]pentixafor (**14**), [^64^Cu]DOTA-ECL1i (**1**), and [^64^Cu]DOTA-DAPTA-comb (**5**) expanded the boundaries of cancer and cardiac disorder imaging for CXCR4, CCR2, and CCR5, respectively. In addition, [^64^Cu]Cu@CuO_x_-ECL1i-Gem [103] and [^177^Lu]pentixather [146] may aid the development of suitable theranostic agents for the treatment of chemokine-related cancers. It is important to note that antibody-based PET radiotracers (immunoPET) have garnered attention in recent years because of their superior target specificity and ability to selectively image immune cells and inflammatory processes [147]. However, their development requires genomic, proteomic, and biological approaches to identify antigens highly or solely expressed on cells, such as tumor cells and immune cells. A couple of Zr-89-labeled antibodies have been reported for the detection of chemokine receptors, such as CXCR4 and CXCR7 in xenograft models in rodents [144,148,149]. However, further studies are needed to translate these into the clinic.

So far, there has been limited success in the development of small-molecule radiotracers for chemokine receptors. Small-molecule radiotracers are advantageous over peptide-based radiotracers as they can penetrate the target tissues under various pathological conditions. Physiological barriers such as endothelial cells and the extracellular matrix lose their structural integrity during the progression of cancer and inflammatory disorders [150,151,152]. When these barriers are intact, small-molecule radiotracers of chemokine receptors could be more efficient in detecting certain inflammatory disorders and cancer in the early phases. Furthermore, literature findings underscore the importance of chemokine receptors, for example, CX3CR1, as promising neuroinflammatory targets [153]. Under such conditions, properly designed small-molecule radiotracers would have the potential to overcome the BBB and would enable the detection of the specific receptors in brain regions.

Despite the success in PET imaging of CXCR4, CCR2, and CCR5 receptors, potential PET radiotracers for other chemokine receptors still need to be developed. Since each chemokine receptor mediates unique and versatile functions on their host cells, their specific PET radiotracers are of importance for target engagement studies in health and disease. Various classes of inhibitors for different chemokine receptors have been developed by independent research groups and global pharmaceutical companies [154,155]. Based on these findings, novel radiotracers can be expected to emerge in the near future. In addition to detecting receptor expression changes in cancer and cardiac and pulmonary disorders, receptor-specific radiotracers would also serve as useful tools in investigating the signal transductions and complex networks of chemokines and chemokine receptors. Depending on the cell or tissue environment under physiological and pathological conditions, a single chemokine can activate multiple receptors and lead to distinct downstream cell signals [156,157]. Receptor-specific PET radiotracers can be exploited to study such signaling mechanisms, which can in turn improve the prognosis of various inflammatory diseases and cancer and thereby provide a path towards the development of more sophisticated and suitable therapeutic strategies.

## Figures and Tables

**Table 1:** Chemokine receptors and their associated key immunoregulatory functions, diseases, and PET radiotracers.

Receptor	Interacting Chemokines	Key Immune Functions	Associated Diseases (Models Studied)	PET Tracer	Ref
CCR1	CCL 3, 5, 8, 14, 15, 16	Macrophage, Natural Killer (NK) cell migration	Multiple myeloma, Rheumatoid Arthritis (RA)	-	[4,5,6,7,8]
CCR2	CCL 2, 7, 8, 13, 16,	Monocyte/Macrophage recruitment	RA, Pulmonary allergy, Atherosclerosis	**1**–**3**	[9,10,11,12,13]
CCR3	CCL 5, 7, 8, 11, 13, 15, 23	Eosinophil distribution and trafficking	Asthma, Allergic rhinitis	-	[14,15]
CCR4	CCL 17, 22	Immune response of T helper type 1, 2 (Th1, 2) cells	Various Carcinomas, Asthma	-	[16,17,18,19,20,21]
CCR5	CCL 3, 4, 5, 7, 13	Th2 cell immune response, Stimulation of T-dendritic cells (DC) interaction, T cell, monocyte, neutrophil recruitment	Breast cancer metastasis, AIDS, Atherosclerosis	**4, 5**	[22,23,24,25,26,27]
CCR6	CCL 20, 21	Migration and recruitment of DC and T cells	Colorectal malignancy, Crohn’s disease	-	[28,29,30,31,32]
CCR7	CCL 19, 21	Stimulation of DC maturation, B, T cell activation	Non-small cell lung cancer, gastric carcinoma	-	[33,34,35,36,37]
CCR8	CCL 18	Monocyte chemotaxis	Allergy, Multiple Sclerosis (MS)	**6**–**8**	[38,39,40]
CCR9	CCL 25	Thymocyte recruitment and development in gut	Inflammatory Bowel Disease (IBD)	-	[41]
CCR10	CCL 27, 28	T cell homing to skin	Skin inflammation	-	[42,43]
CXCR2	CXCL 1, 2, 3, 5	Neutrophil trafficking	Chronic Obstructive Pulmonary Disease (COPD)	-	[44]
CXCR3	CXCL 9, 10, 11	NK, Th1, and CD8+ T cell trafficking and immune response	Pulmonary fibrosis, Atherosclerosis, Multiple Sclerosis	-	[45,46,47,48,49,50]
CXCR4	CXCL 12	Lymphocyte chemotaxis	Various cancers, AIDS	**9–29**	[43,51,52]
CXCR5	CXCL 13	B cell migration	Breast cancer, MS	-	[53,54,55,56]
CXCR7	CXCL 11, 12	Recruitment of β-arrestins	Breast and lung squamous cell cancer	**30**	[57,58,59]
CX3CR1	CX3CL1	Lymphocytes and leukocytes migration	Neuroinflammation	**31**	[60,61,62,63]

## Data Availability

Not applicable.
